# Divergent Mentalization Types in Adolescent Borderline Personality Disorder and Attention Deficit/Hyperactivity Disorder

**DOI:** 10.1192/j.eurpsy.2023.793

**Published:** 2023-07-19

**Authors:** O. F. Akca, K. Wall, C. Sharp

**Affiliations:** 1 Child and Adolescent Psychiatry, Necmettin Erbakan University Meram School of Medicine, Konya, Türkiye; 2 Psychology, University of Houston, Houston, TX, United States

## Abstract

**Introduction:**

Attention Deficit/Hyperactivity Disorder (ADHD) and Borderline Personality Disorder (BPD) have several similarities and it is difficult to distinguish these disorders in adolescents.

**Objectives:**

We aimed to identify the unique correlates of mentalization abilities that may distinguish these two disorders, and to investigate the mentalization abilities of adolescents with ADHD, BPD and ADHD+BPD in an inpatient sample (n=550) to determine the effect of co-morbidity on mentalization abilities.

**Methods:**

We have explored the relationship between Child Eye Test (CET) scores, Movie for the Assessment of Social Cognition (MASC) subscales, and ADHD and BPD symptoms in adolescent inpatients. In addition, we compared ADHD, BPD and ADHD+BPD groups in terms of their mentalization abilities.

**Results:**

Correct MASC scores were negatively associated with both ADHD and BPD symptoms in girls, and negatively associated with ADHD symptoms in boys. In addition, hypermentalization scores were associated with BPD symptoms in girls, and hypomentalization and no mentalization scores were associated with ADHD symptoms in girls. CET scores were negatively associated with ADHD symptoms in girls, but no relations with BPD were found. Group comparisons revealed no significant difference among groups.

**Conclusions:**

We found that while ADHD symptoms are related to hypomentalization, BPD symptoms are rather related to hypermentalization. We believe that these findings make significant contributions to literature aimed at understanding the differences between two disorders which have great commonalities in terms of clinical appearance and developmental course.

**Disclosure of Interest:**

None Declared

